# Mechanisms Underlying the Anti-Suicidal Treatment Potential of Buprenorphine

**DOI:** 10.3389/adar.2021.10009

**Published:** 2021-08-03

**Authors:** Courtney M. Cameron, Steven Nieto, Lucienne Bosler, Megan Wong, Isabel Bishop, Larissa Mooney, Catherine M. Cahill

**Affiliations:** 1 Department of Psychiatry and Biobehavioral Sciences, University of California, Los Angeles, Los Angeles, CA, United States; 2 Shirley and Stefan Hatos Center for Neuropharmacology, University of California, Los Angeles, Los Angeles, CA, United States; 3 Semel Institute for Neuroscience and Human Behavior, University of California, Los Angeles, Los Angeles, CA, United States; 4 David Geffen School of Medicine, University of California, Los Angeles, Los Angeles, CA, United States; 5 Department of Psychology, University of California, Los Angeles, Los Angeles, CA, United States

**Keywords:** suicide, suicidal ideation, kappa opioid, addiction, pain, opioid use disorder, anhedonia, dysphoria

## Abstract

Death by suicide is a global epidemic with over 800 K suicidal deaths worlwide in 2012. Suicide is the 10th leading cause of death among Americans and more than 44 K people died by suicide in 2019 in the United States. Patients with chronic pain, including, but not limited to, those with substance use disorders, are particularly vulnerable. Chronic pain patients have twice the risk of death by suicide compared to those without pain, and 50% of chronic pain patients report that they have considered suicide at some point due to their pain. The kappa opioid system is implicated in negative mood states including dysphoria, depression, and anxiety, and recent evidence shows that chronic pain increases the function of this system in limbic brain regions important for affect and motivation. Additionally, dynorphin, the endogenous ligand that activates the kappa opioid receptor is increased in the caudate putamen of human suicide victims. A potential treatment for reducing suicidal ideation and suicidal attempts is buprenorphine. Buprenorphine, a partial mu opioid agonist with kappa opioid antagonist properties, reduced suicidal ideation in chronic pain patients with and without an opioid use disorder. This review will highlight the clinical and preclinical evidence to support the use of buprenorphine in mitigating pain-induced negative affective states and suicidal thoughts, where these effects are at least partially mediated via its kappa antagonist properties.

## INTRODUCTION

Suicide is a pressing public health issue that accounts for more than 800,000 deaths per year globally [1]. In the United States, it is one of the 10 leading causes of death claiming more than 47,000 lives in 2019, and the second leading cause of death in people aged 10 to 34 [2]. The rates of suicide have risen more than 30% between 1999–2019. The United States Surgeon General and Department of Health and Human Services have recently issued a call to action to implement the National Strategy for Suicide Prevention that includes calls for treatment access to those that need it [3]. Important, for this review, is that chronic pain is second only to bipolar disorder as the major cause of suicide among all medical illnesses [4, 5]. Suicide is typically preceded by suicidal ideation–persistent thoughts about wanting to kill oneself. While suicidal ideation only rarely leads to the completion of suicide (death), it is an important clinical marker, and prior suicide attempt is the single strongest risk factor for future suicidal behaviors and death by suicide [6].

Recently, there has been a dramatic increase in the prevalence of suicidal ideation during the COVID-19 pandemic and subsequent mitigation activities, including social distancing and stay-at-home orders [7, 8]. In June 2020, 10.7% of adults reported thoughts of suicide compared to 4.3% of adults in 2018 [9]. Fortunately, suicide rates themselves have remained unchanged or declined early in the pandemic [10]. However, the management of patients suffering from suicidal ideation represents a rapidly growing clinical challenge. There are currently few pharmacological treatments available that specifically alleviate suicidal ideation; therefore, research into this clinical problem has the potential to save many lives.

Suicidal ideation occurs in a subset of individuals with major depressive disorder (MDD); however, emerging evidence suggests that suicidal ideation may also represent a distinct behavioral disorder [11]. For example, improvements in suicidal ideation following ketamine treatment cannot be entirely explained by improvements in depression or anxiety [12]. Suicidality also often presents with mental health disorders other than depression. Substance use is a risk factor for suicide attempts, and individuals with substance use disorders have a 10–14 times greater risk of death by suicide compared to the general population [13-15]. Many substance overdoses may also be unrecognized suicides [16]. Some studies have demonstrated that anxiety disorder comorbidity with other mood disorders is a risk factor for suicide attempts, though this finding has been inconsistent [17]. The comorbidity of borderline personality disorder and depression is associated with an increase in the number and seriousness of suicide attempts [18].

Acute and chronic alcohol use also play major roles in suicidal behavior. Approximately 26% of suicide decedents who were tested for alcohol had intoxicating blood alcohol levels (>0.08%) [19]. According to recent meta-analyses, which include case-control and cohort studies, individuals with alcohol use disorder (AUD) have three times greater odds of suicidal behavior compared to those without the disorder [20, 21]. Furthermore, AUD has been identified as the second most common mental disorder among suicide decedents [22].

Finally, suicidal ideation is highly prevalent in chronic pain patients [23-26], even when controlling for the subjective severity of pain symptoms [27-29] and the presence of other affective disorders [27, 30, 31]. For such patients, suicide is viewed as a means to alleviate overwhelming and intolerable painful internal states, and psychological and emotional pain have been considered essential for suicidal behavior [32, 33]. A recent metanalysis of 31 studies showed a significant link between physical pain and suicidal thoughts and behaviors, where physical pain was associated with lifetime death wish, current and lifetime suicidal ideation, suicide plan, and suicide attempts, as well as death [34]. Some of the predictors of suicidal ideation in chronic pain patients include mental defeat [35], insomnia [36], and pain catastrophizing [37], which are also risk factors for opioid use disorder. Suicidal ideation in chronic pain patients is associated with depression, anxiety and sleep disorders [38]. This review will highlight recent data that provides justification for further clinical trials to test the potential of buprenorphine as a treatment for suicidal ideation, particularly in chronic pain patients.

### The Affective (Emotional) Dimension of Pain and Suicidal Ideation

Chronic pain has both sensory and emotional/affective components that, while distinct, share many of the same neurobiological substrates. Chronic pain often induces a persistent negative affective state, or “emotional pain,” likely as a result of neuroadaptations in the brain’s reward processing circuitry [39-41]. Chronic pain is also associated with the development of other disorders of impaired reward processing, including depression and substance use disorders [42]. The prevalence of suicidal ideation among pain patients suggests that it may be an expression of emotional pain. In fact, studies have found emotional pain to be the psychological variable most strongly associated with current suicidality, even more so than the presence of depressed mood or hopelessness [18, 43, 44].

The relationship between AUD and suicidality also suggests that suicidal ideation may be an expression of emotional pain. That AUD is often a key risk factor for suicidal behavior is likely due, at least in part, to alcohol-induced exacerbations in negative emotionality and alcohol-related negative consequences, particularly in interpersonal domains [45]. Hyper-negative emotional states and hyperalgesia are both consequences of repeated alcohol use [46] and may contribute to an increase in alcohol use as a compensatory mechanism [47]. Among heavy drinkers, the negative emotional components of pain (i.e., pain catastrophizing) can enhance alcohol craving more than physical pain [48].

The emotional pain caused by the disruption or loss of social attachments also plays an important role in suicidality. In particular, suicidal ideation shares neurobiological and psychological features with separation distress—the innate, emotionally painful, dysphoric response of animals and humans to social separation or rejection [49, 50]. Converging evidence from preclinical and clinical studies supports the link between suicidal ideation and separation distress. Opioids reduce separation distress behaviors in non-human mammals [51-53] and have recently shown promise for the treatment of suicidality [54, 55]. Suicidal acts are most common after interpersonal losses or rejections [56], and patients with borderline personality disorder are particularly susceptible to social rejection and often become suicidal after interpersonal rejections [57, 58].

### An Affective Neuroscience Model Linking Pain, Suicidal Ideation, and Depression

Research in the field of affective neuroscience suggests that separation distress represents one of the ancestral primary-process emotional systems (referred to as PANIC/GRIEF) [49, 50, 59, 60]. Importantly, the PANIC/GRIEF system probably evolved from general pain mechanisms [59], and studies have found a link between separation distress and physical pain. Maternal separation in mouse neonates is capable of altering nociceptive behavior in adulthood [61, 62]. In humans, early life adversity in the form of both physical and psychological trauma (including familial separation) is associated with an increased risk of chronic pain in later life [63]. Studies show that parental bonding in adolescents is significantly associated with adolescent chronic pain and depression, where low maternal care contributes to increased pain via heightened depressive symptoms [64].

The neuroanatomy of the PANIC/GRIEF system overlaps with the brain’s system for processing physical pain [65], suggesting that both physically painful (e.g., injury) and emotionally painful (e.g., interpersonal rejection) stimuli may engage this shared neurocircuitry to produce distress and dysphoria (emotional pain), with separation distress representing a particular subtype of emotional pain. The PANIC/GRIEF system also represents a potential substrate linking emotional pain/distress with suicidal ideation. The similarities between suicidal ideation and separation distress discussed above suggest that suicidal ideation may result from increased activity in the brain’s PANIC/GRIEF network, whether initiated by social loss or physical injury. Thus, chronic pain patients may be particularly susceptible to suicidal ideation due to persistent activation of this system by painful sensory stimuli.

Sustained activation of the PANIC/GRIEF system can also lead to reduced activity in the brain reward SEEKING system (the system primarily responsible for motivation and arousal, particularly the mesolimbic dopamine pathway), perhaps as a means to protect against sustained emotional pain by reducing the overall arousal of emotions [49, 50, 59, 66]. Underactivity in the SEEKING system causes blunted reward processing, leading to an amotivational state characterized by the diminished experience of positive feelings (anhedonia). Thus, down-regulation of the SEEKING system in response to sustained emotional pain (dysphoria) may be one underlying cause of the anhedonia and blunted affect that is characteristic of depression.

**FIGURE 1 ∣:**
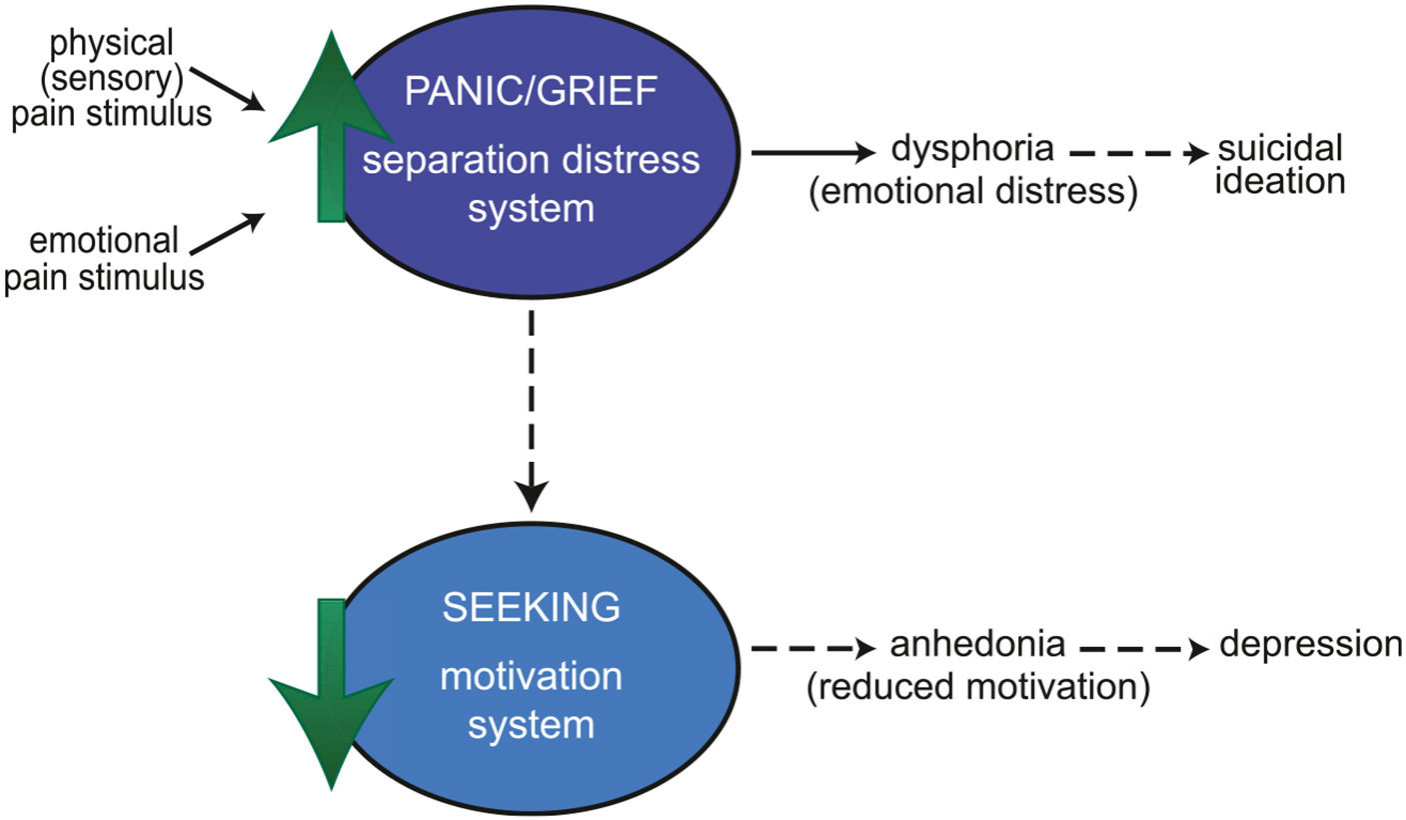
An affective neuroscience model linking pain, suicidal ideation, and depression. Both physical (sensory) pain (such as injury) and emotional pain (such as interpersonal rejection) stimuli can act on the PANIC/GRIEF (separation distress) system. An increase in activity in this system produces dysphoria (emotional distress). Sustained activation of the PANIC/GRIEF system (dotted lines) could lead to suicidal ideation and a reduction in downstream activity of the SEEKING (motivation) system. Reduced motivation (anhedonia) may then result in loss of interest in activities characteristic of depression. This model suggests that suicidal ideation would often be comorbid with depression but could also occur independently of a disruption in reward processing.

This two-stage model then suggests that the anhedonia of depression may reflect, in part, an emotional shutdown that follows the behavioral agitation of separation distress or other emotional pain, with these behavioral states correlating with underactivity of the SEEKING network and overactivity of the separation distress PANIC/GRIEF network, respectively. Of course, depression is a complex disorder that likely has multiple etiologies involving changes in many neural substrates. However, this model suggests one possible framework for understanding the relationship between suicidal ideation and depression. Sustained activation of the PANIC/GRIEF network may produce both a state of dysphoria that leads to suicidal ideation as well as, indirectly, a reduction in motivation (anhedonia) through subsequent downregulation of the reward SEEKING system (Figure 1). In this way, suicidal ideation would often be comorbid with depression but could be the result of dysregulation of the PANIC/GRIEF circuitry independent of a disruption in reward processing. In support of this hypothesis, patients with borderline personality disorder, but not comorbid depression, typically experience brief durations of suicidality as a result of interpersonal stresses. In contrast, patients with comorbid borderline personality disorder and depression display more persistent symptoms of depression and suicidality, which also include a loss of interest in ordinarily pleasurable activities (anhedonia) [18].

Ultimately, painful experiences (whether the emotional pain of social rejection or the physical/sensory pain characteristic of chronic pain conditions) likely engage shared brain systems which produce negative affective states (distress/ dysphoria–implicated in suicidal ideation) and may subsequently disrupt downstream reward processing (anhedonia–implicated in depression), leading to a complex relationship between pain, separation/social distress, and motivation [65, 67, 68]. The neurocircuitry and neurochemistry underlying each is discussed in more detail below.

### Neurocircuitry of Pain, Separation/Social Distress, and Motivation

#### Acute and Chronic Pain

Pain is a multidimensional experience comprised of sensory, cognitive, and emotional components. The sensory aspects of pain are relayed from peripheral nociceptors, which detect noxious sensory stimuli, along primary afferent neurons that have central terminals in the spinal cord [69]. The second order neurons in the spinal cord ascend to various brain structures including the nucleus of the solitary tract, the medial brain stem reticular formation, the caudal ventrolateral medulla, the lateral parabrachial nucleus, the midbrain periaqueductal gray, and the thalamus and the hypothalamus [69, 70]. The classical pathways associated with ascending nociceptive information are the lateral and medial spinothalamic pathways, where the lateral is responsible for sharp, well-localized pain and the medial is for diffuse, poorly localized persistent pain. Sensory information such as pain intensity and location (discriminative aspects of pain) is then relayed along the lateral spinothalamic tract to the primary and secondary somatosensory cortices via the medial thalamic nuclei. A descending pain modulation system originating in the periaqueductal gray (PAG) also regulates pain signals at the level of the spinal cord before they are relayed to higher subcortical and cortical structures [71].

**FIGURE 2 ∣:**
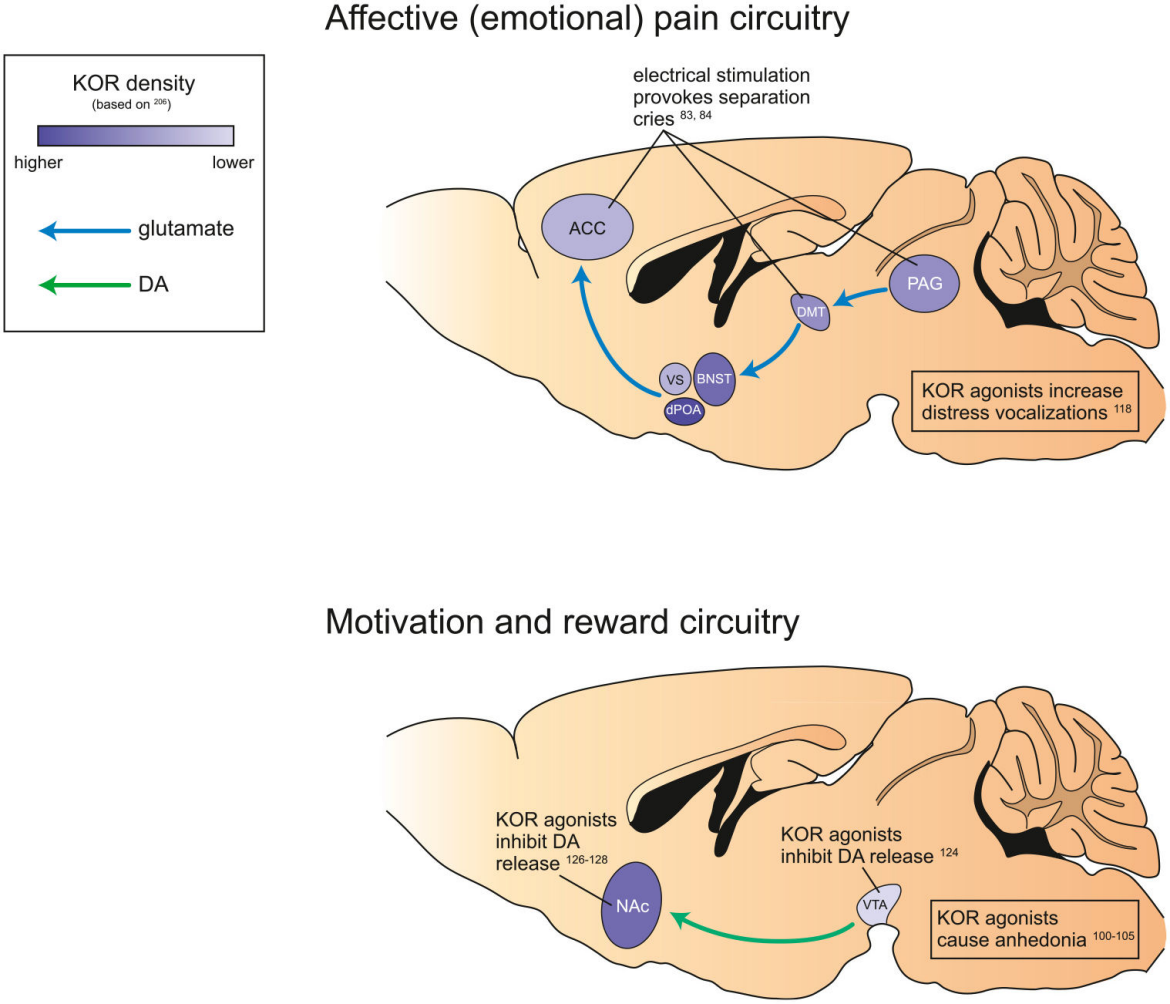
Neurocircuitry and kappa opioid signaling involved in affective (emotional) pain and motivation and reward. Schematic of brain areas implicated in affective (emotional) pain, particularly separation distress **(top)**, and motivation and reward **(bottom)** in rodents. Both circuits are altered by activity at KOR (present to varying degrees in all relevant brain regions). KOR antagonism increases distress vocalizations, inhibits mesolimbic DA release, and causes associated dysphoria and anhedonia. anterior cingulate cortex, ACC; ventral septum, VS; dorsal preoptic area, dPOA; bed nucleus of the stria terminalis, BNST; dorsalmedial thalamus, DMT; periaqueductal gray, PAG; nucleus accumbens, NAc; ventral tegmental area, VTA.

The emotional and motivational aspects of pain (e.g., subjective unpleasantness and salience) are carried to limbic structures such as the amygdala, hypothalamus, striatum, insula, and anterior cingulate cortex by the medial spinothalamic tract [72-74] (Figure 2). A key structure for encoding the affective component of pain is the parabrachial complex located in the pons, receiving dense inputs form lamina I nociceptive spinal neurons; a projection reportedly denser than the spinothalamic pathway [75, 76]. The parabrachial complex projects to several regions involved in pain and affect including the PAG, rostroventral medulla, thalamus, amygdala and zona incerta, making it a key structure for the affective emotional perception of pain.

The anterior cingulate has also been shown to be a critical brain region for the modulation of the subjective affective experience of pain [73]. Patients who have had a portion of the anterior cingulate surgically removed report that painful stimuli are no longer bothersome, even though they are able to localize pain sensations [77]. These findings support the idea that the distressing affective experience of a physically painful stimulus can be separated from its sensory properties.

Emotional pain also activates brain regions associated with physical pain including the PAG, insula, and anterior cingulate [78]; similarly, physical pain also activates limbic structures including the nucleus accumbens, ventral tegmental area (VTA), amygdala, and habenula [79-81]. Thus, the sensory and emotional components of pain are processed within discreet but interacting brain structures.

While a painful event serves an adaptive function and provides salience to a harmful stimulus that can support escape and avoidance learning, chronic pain can become pathological (serving no useful purpose). Although, it was recently proposed that this type of pain perhaps serves to provide hypervigilance [82]. Chronic pain involves neuroplasticity in the circuitry underlying both the sensory and affective components of pain. Patients with chronic pain suffer from sensory disturbances including allodynia (pain caused by a previously nonpainful stimulus) and hyperalgesia (exaggerated pain response to a previously painful stimulus). However, the negative affective component of chronic pain is argued to be a greater factor in quality of life measures [40].

### Separation/Social Distress

Much of the same neurocircuitry described above is also engaged by separation distress, suggesting that the emotional pain elicited by separation distress is a result of the activation of circuits underlying physical pain. Specifically, the separation distress circuitry starts in the PAG and ascends through the dorsomedial thalamus, terminating in various basal forebrain regions including the anterior cingulate cortex [50, 59, 65] (Figure 2). Localized electrical stimulation of the anterior cingulate, dorsomedial thalamus, and PAG provoke separation cries in mammals [83, 84]. In humans, the experience of emotional distress induced by social rejection is associated with an increase in activity in the anterior cingulate cortex measured by fMRI [85]. Together, these studies suggest that psychological pain, particularly social rejection and intense loneliness, may share some of the same neural pathways that elaborate physical pain.

### Motivation

Reward and motivation, while often considered opponent processes to pain, are also processed within many of the same brain structures, in particular the mesolimbic system, which includes the VTA and nucleus accumbens (Figure 2). In neuropathic pain animals, functional connectivity is altered within the limbic system (including the nucleus accumbens) as well as between the limbic and nociceptive systems (including the thalamus, primary sensory cortices, insula, and PAG) [86]. In human clinical pain cohorts, connectivity is altered between the mesolimbic system and cortical structures [87-89]. Given that the mesolimbic system is responsible for the modulation of motivated behaviors and reinforcement learning [90, 91], altered activity in this system likely contributes to the negative affective component of pain. Pain can also directly impair general reward processing, leading to an anhedonic state [42]. Dopamine is a critical neurotransmitter within the mesolimbic system, and dopamine signaling in the nucleus accumbens may modulate the salience of painful experiences. While acute pain activates dopaminergic transmission to the nucleus accumbens [81, 92], chronic pain produces the opposite effect [93-95].

Together, the circuitry underlying pain, separation distress, and motivation provides a neuroanatomical substrate for the transition from physical or emotional pain to suicidality and possibly depression. Activity in pain/nociceptive circuitry (including the anterior cingulate, dorsomedial thalamus, and PAG) is correlated with the distress PANIC/GRIEF system, while activity in the mesolimbic circuit, particularly dopamine transmission in the nucleus accumbens, is correlated with the SEEKING system [50, 60, 66]. Sustained activity in the pain circuitry can reduce activity in the motivational circuitry through modulation of mesolimbic dopamine signaling, such that the distress caused by prolonged pain (sensory or emotional) may ultimately lead to decreased processing of rewarding stimuli. These neural substrates are further linked through shared activation by the opioid system, discussed in more detail below.

### The Opioid System

#### Receptors and Endogenous Ligands

The opioid system, which modulates pain, social distress, and reward circuitry, may be a promising target for the treatment of behavioral disorders caused by disruptions in this circuitry, including suicidal ideation. Opioid receptors belong to the G-protein coupled receptor family and are divided into four families: the mu (MOR), delta (DOR), kappa (KOR) and nociceptin (NOR). These receptors are activated by four classes of endogenous opioid peptides, beta-endorphin, dynorphin, enkephalin and nociceptin. MORs have a high affinity for beta-endorphin and enkephalins, but low affinity for dynorphin. Conversely, dynorphin primarily acts through the KOR. Here we focus on the role of the MOR and, particularly, the KOR, as both have been implicated in the mediation of suicidal ideation by opioid drugs.

Opioid receptors are distributed throughout the central and peripheral nervous system and are present in many of the major structures involved in the pain circuitry, including the peripheral nociceptors, spinal cord, PAG, thalamus, anterior cingulate cortex, and other limbic regions [96]. The MOR is widely distributed throughout the brainstem, midbrain, and forebrain structures, and mediates the analgesic effects of most clinically available opioid medications, such as morphine [97]. KORs are located throughout the neuroaxis as well, and their localization in the spinal cord and brain stem can produce analgesia through the direct inhibition of pain pathways [98, 99].

While KOR and MOR expression widely overlaps throughout the brain, their activation produces opposing effects on mood [100]. Activation of KORs primarily produces negative emotions and dysphoria [101, 102], including depressive-like and psychotomimetic effects in humans [103-105] and rodents [100, 106-109]. In contrast, activation of the MOR is reinforcing and associated with positive hedonic experiences, Thus, in general, KOR activity is involved in an anti-reward system opposing rewarding MOR activity [110].

### Involvement in Pain, Separation/Social Distress, and Reward/Motivation

Opioids and their receptors can modulate both the sensory and emotional (bothersome) components of pain. For example, when injected into the dorsomedial thalamus (a key region for processing both the sensory and affective components of pain), the MOR agonist DAGO elevated rats’ sensory pain thresholds and induced a positive affective state, while the KOR agonist U50,488 reduced rats’ pain thresholds and induced a negative affective state [111]. However, studies have shown sex differences with respect to the functioning of the MOR and KOR systems, particularly with respect to pain and addiction. Several comprehensive reviews are available [112-114].

In addition to their role in the sensory/affective components of pain, it is hypothesized that opioids constitute a major neurochemical underpinning of social bonding and isolation distress [115, 116]. For example, opioid peptides are decreased in the midbrain of rat pups following social isolation [117], and endogenous opioid peptides acting at MORs have been shown in animal models to alleviate distress behaviors following social separation [115]. The MOR agonist morphine decreases distress vocalizations in rat pups isolated from their mother [118, 119], while the KOR agonist U50,488 increases isolation-induced ultrasonic vocalization [118]. Thus, it appears that dynorphins are responsible for mediating negative affect within the neurocircuitry underlying social distress, similar to their role in the modulation of physical pain (Figure 2).

High expression levels of KOR have been also detected in brain areas responsible for reward and motivation, including the VTA and nucleus accumbens [120, 121] (Figure 2). The ability of KOR agonists to negatively modulate mesolimbic dopamine has significant implications for motivated behavior. Since reduced nucleus accumbens dopamine signaling is associated with a loss of motivation, KOR modulation of dopamine circuitry may link the acute distress of physical or emotional pain with the subsequent onset of negative motivational states and affect.

Studies in rat brain slices show that KOR agonists (U69,593) are capable of suppressing mesolimbic dopamine release via receptors expressed on dopamine neuronal terminals as well as neuronal cell bodies [122-125], which may contribute to the dysphoric effects of KOR activation. KOR agonists (including U50,488, spiradoline, U69,593) also inhibit dopamine signaling when applied directly into the nucleus accumbens of rats, as measured by microdialysis in intact animals [126, 127] or by superfused brain slices [128]; however, changes in dopamine signaling in the nucleus accumbens did not correlate with KOR agonist (U50,488) induced conditioned place aversion (CPA) in mice [129]. Furthermore, morphine-evoked increases in extracellular dopamine within the nucleus accumbens were blocked by the administration of a KOR agonist (U50,488) into this brain region in mice [130]. Finally, the expression of KOR-mediated aversion (U69,593-induced CPA) requires the activity of medium spiny neurons expressing dopamine receptors within the nucleus accumbens of rats [131] and mice [132].

Activation of KORs also contributes to the dopamine hypofunction observed in chronic pain states. Given that hypo-dopaminergic states contribute to chronic pain [133, 134] and mood disorders comorbid with chronic pain [135], KOR antagonism to recover dopamine may hold promise as a novel therapeutic for treating chronic pain and associated mood disorders. Indeed, reduced motivation for food (sucrose) reward induced by inflammatory pain was recovered by KOR antagonism (with norBNI) or silencing of dynorphin neurons within the ventral striatum of rats [136]. A comprehensive review of KOR function in chronic pain and its relationship with drugseeking behavior is available [137].

The role of KORs in dysfunctions of reward and motivation is particularly well-characterized with respect to AUD. KOR-mediated reductions in dopamine release in the nucleus accumbens have been hypothesized to mediate negative emotional states associated with alcohol withdrawal, particularly pain associated with acute alcohol withdrawal [138]. At the preclinical level, alcohol-preferring rats show increased dynorphin mRNA expression in the central amygdala and hypothalamus compared to non-preferring rats after voluntary drinking [139]. The KOR antagonist norBNI also attenuates withdrawal-related anxiety-like behaviors in alcohol-dependent mice [140]. In addition to alleviating alcohol-induced negative affective states, pharmacotherapies with KOR antagonist properties, including buprenorphine, reduce binge-like alcohol drinking [141, 142], alcohol self-administration [143, 144], and block escalation of compulsive-like drinking after dependence induction [145, 146] and stress exposure [147] in rodent.

The ability of opioids to modulate both sensory/affective pain circuits as well as reward/motivation circuits suggests these compounds have the capability to counteract multiple features of suicidality and depression. Opioids are able to produce dopamine-independent positive affective states through their influence on pain and social distress circuits, including areas such as the anterior cingulate cortex, PAG, and dorsomedial thalamus. Opioids can also counteract negative affective states by promoting increased motivational drive through their downstream influence on dopaminergic reward circuits, especially in the nucleus accumbens. Thus, since opioids can restore deficits in both pain and reward circuits, they may be particularly useful in treating suicidal ideation, as they would be able to blunt suicidality whether it was caused primarily by a disruption in dopaminergic motivational circuity or a disruption in non-dopaminergic pain/social distress circuits. Other treatments which primarily target reduced motivation (anhedonia) may not be effective in treating the subset of individuals experiencing suicidal ideation without a concomitant reduction in motivation (i.e., patients with or without comorbid depression).

### Buprenorphine Attenuates Suicidal Ideation

Given the above understanding of the opioid system, there has been a renewed interest in the use of opioid analgesics, particularly buprenorphine, for the treatment of suicidality. A retrospective 24-months study reported buprenorphine had the lowest incidence of suicide intent and deaths compared to other opioid analgesics [148]. Buprenorphine has demonstrated rapid antidepressant effects in humans [149-152], including those with treatment-resistant depression, and has shown particular promise in reducing suicidal ideation [153].

### Buprenorphine Mechanism

Buprenorphine is an analgesic derived from oripavine that acts as a partial MOR agonist and KOR antagonist, as well as an antagonist at the DOR and an agonist at the NOR [154]. Buprenorphine has similar affinities for the MOR and KOR, but a 10-fold lower affinity for the DOR [154]. For the purposes of this review, we have largely limited our discussion to the MOR agonist and KOR antagonist effects as these are well-characterized, although note that some work has shown that buprenorphine may have partial agonist activity at KORs [155-157]. Importantly, due to its partial MOR agonist properties, buprenorphine has a lower overdose risk compared to full MOR agonists such as morphine [158].

### Clinical Studies Demonstrating the Effects of Buprenorphine on Behavior

The potential anti-suicidal effects of buprenorphine were first described in a case report of a 61-year-old woman suffering from treatment-resistant depression, chronic back pain, severe opioid use disorder, and ongoing suicidal ideation [159]. While treatment with buprenorphine (16 mg/4 mg buprenorphine/naloxone) was prescribed to treat the patient’s opioid use disorder, she reported that her suicidal ideation completely disappeared after 1 week of treatment, and suicidal ideation remained absent up to 3 months after the initial treatment. In another case report, a patient with cannabis-induced psychotic disorder and opioid depressive disorder with severe suicidal thoughts was treated successfully with a single high dose (96 mg) of buprenorphine [160]. Another case report showed that buprenorphine/naloxone (8 mg/2 mg) was effective in reducing pain and suicidal ideation in a 39-year-old male with a history of bipolar disorder, multiple suicide attempts, and polysubstance abuse [161]. Chart reviews of suicidal adult depressed patients with comorbid chronic pain and opioid use disorder who received off-label buprenorphine also found some support for the anti-suicidal properties of buprenorphine [162]. The presence of chronic pain, depression, and substance use in these case reports and studies suggests that buprenorphine may effectively mitigate suicidal ideation by targeting multiple overlapping neurocircuits, which underlie these often-comorbid disorders.

Based on these case reports and anecdotal findings, clinical trials have begun to explore the anti-suicidal potential of buprenorphine. A multisite randomized double-blind placebo-controlled trial of ultra-low-dose (initial dosage, 0.1 mg once or twice daily; mean final dosage, 0.44 mg/day) buprenorphine found that severely suicidal patients showed a reduction in Beck Scale for Suicidal Ideation (BSSI) scores after 2 and 4 weeks of treatment compared to patients that received placebo [54]. Another randomized clinical trial tested the efficacy of one of three single high doses of buprenorphine (32, 64, 96 mg) in suicidal opioid-dependent patients [163]. The researchers found that BSSI scores were significantly reduced in patients across all three buprenorphine doses. While these findings suggest that buprenorphine has particular promise as an anti-suicide treatment option, more research is needed to determine the conditions under which buprenorphine treatment is most effective and tolerable, including whether low or high doses are more successful (the wide range of effective doses in these clinical trials - from 0.44 to 96 mg - is interesting and will be an important area for future studies) and whether treatment is affected by comorbidities including substance use disorder or chronic pain.

Other studies have examined the effects of buprenorphine in combination with other drugs. A randomized double-blind placebo-controlled trial in adults with treatment-resistant depression showed that treatment with 2 mg/2 mg buprenorphine/samidorphan significantly improved scores on multiple depression measures compared to placebo controls [164]. Antidepressant activity was also demonstrated in another study using a 1:1 ratio of buprenorphine:samidorphan [165]. Samidorphan is a MOR antagonist, thus this combined treatment leaves the KOR antagonist activity of buprenorphine intact while blocking the MOR agonist activity of buprenorphine. Blocking the subjective and objective MOR effects likely mitigates the potential addictive properties of buprenorphine in opioid-naïve individuals. While these studies did not directly measure suicidal ideation independent of depression, they provide important insight into the potential mechanisms of buprenorphine’s behavioral effects. In both cases, the effects of buprenorphine were not blocked by a MOR antagonist, suggesting that activity at this receptor may not be required for the anti-suicidal effects of buprenorphine.

### Preclinical Studies Demonstrating the Effects of Buprenorphine on Behavior

In addition to the clinical findings described above, pre-clinical studies also support the potential anti-suicidal treatment effects of buprenorphine. While no behavioral assays for suicidal ideation exist for rodents, several behavioral assays have been validated to screen for depressive-like behaviors, including the forced swim test and the novelty-induced hypophagia test. The forced swim test is considered a measurement of behavioral despair [166] and is one of the gold-standard screens for depressive behavior as it is reliably reversed by antidepressants [167]. The novelty-induced hypophagia test is a conflict-based behavioral task that assesses the impact of an environmental stressor on animals’ conditioned approach toward a palatable food reward, with longer approach latencies indicating greater depressive or anxious behavior [168]. Treatment with antidepressants or benzodiazepines reduces approach latencies in the novelty-induced hypophagia test [168-170].

In mice, administration of buprenorphine produced significant reductions in forced swim test immobility (at doses ranging from 0.065–2 mg/kg) and reduced approach latencies in the novel environment of the novelty-induced hypophagia test (at a dose of 0.25 mg/kg) [171]. In Wistar Kyoto (WKY) rats, a strain which has an exaggerated depressive phenotype and is resistant to certain antidepressants, buprenorphine (2.25 mg/kg) significantly reduced immobility in the forced swim test [172]. This effect was specific to the WKY strain, suggesting that buprenorphine may be more effective in individuals with certain treatment-resistant depressions. In general, the doses which produced behavioral effects in these preclinical studies were lower than those used in clinical trials; however, one trial showed that an ultra-low dose of buprenorphine (0.44 mg/day, comparable to the preclinical doses) significantly reduced suicidal ideation [54].

### The Contribution of Kappa Versus Mu Activity in the Anti-Suicidal Effects of Buprenorphine

A better understanding of the specific pharmacological underpinnings of the anti-suicidal and anti-depressive effects of buprenorphine would be valuable as it could inform the development of even more targeted therapeutics which might avoid side effects, including abuse potential. As discussed previously, buprenorphine is both a partial agonist at the MOR and an antagonist at the KOR, but which receptor type is primarily responsible for the anti-suicidal and anti-depressive effects of the drug is uncertain. A review of preclinical studies can help to shed light on this question. In general, the data are conflicting, and both MOR partial agonist and KOR antagonist activity likely play a role in the anti-suicidal effects of buprenorphine. However, the data supporting the importance of KOR antagonism appear slightly more consistent (discussed in more detail below).

### Studies With Buprenorphine

Studies combining buprenorphine with opioid antagonists suggest that the MOR is not necessary for the behavioral effects of buprenorphine. In mice, the anti-depressive effects of buprenorphine described above (reduction in forced swim test immobility and reduced approach latency in the novelty-induced hypophagia test) were maintained when buprenorphine (1 mg/kg) was co-administered with the opioid antagonist naltrexone (1 mg/kg), suggesting that activation of MORs is not necessary for the expression of buprenorphine’s anti-depressive effects [173]. Co-administration of buprenorphine (0.1 mg/kg) with the MOR antagonist samidorphan (0.3 mg/kg) in Wistar Kyoto rats did not alter the drug’s efficacy in the forced swim test [174], again suggesting that activation of MOR is not necessary for the anti-depressive effects of buprenorphine.

The use of selective knockout mice provides evidence that the behavioral effects of buprenorphine may be mediated by both MOR and KOR. In mice with genetic deletion of the MOR (Oprm1^−/−^) or KOR (Oprk1^−/−^), buprenorphine (0.25 mg/kg)-induced decreases in latency in the novelty-induced hypophagia test were blocked in Oprm1^−/−^ but not Oprk1^−/−^ mice [175], suggesting that buprenorphine’s activity at MOR, but not KOR, is required for its anti-depressive effects. Consistent with this idea, a mouse model of the A118G polymorphism (associated with less opioid receptor expression and lower signaling efficiency) in the MOR gene (OPRM1) also disrupted the effects of buprenorphine on this behavior [176]. These data are further supported by human studies which have found that the A118G polymorphism was associated with treatment onset suicidal ideation [177] and more severe depression following a recent targeted rejection major life event [178].

In contrast, knockout of the KOR in mice blocked buprenorphine’s reduction of immobility in the forced swim test while knockout of the MOR did not disrupt the behavioral effects of buprenorphine (0.25–0.5 mg/kg) [179], suggesting that buprenorphine’s activity at KOR, but not MOR, is required for its anti-depressive effects. Together, these studies indicate that the role of different opioid receptor types in buprenorphine’s anti-suicidal and anti-depressive effects are likely mediated by multiple factors including behavioral assay, genetic background, and drug dose.

### Studies With Other Drugs That Have Kappa and Mu Activity

We can also examine the behavioral effects of other KOR and MOR drugs to further explore the potential role of opioid receptor subtype in the behavioral effects of buprenorphine. Since buprenorphine is an antagonist at the KOR, if activity at this receptor is primarily responsible for the anti-suicidal effects of buprenorphine, we would expect manipulations that increase activity at KOR to be pro-depressive while manipulations that decrease activity at KOR to be anti-depressive (Table 1, dark blue cells). Conversely, buprenorphine is a partial agonist of the MOR, so if activity at this receptor is primarily responsible for the anti-suicidal effects of buprenorphine, we would expect manipulations that increase activity at MOR to be anti-depressive while manipulations that decrease activity at MOR to be pro-depressive (Table 2, yellow cells).

### Kappa Agonists

In general, KOR agonists produce behaviors indicative of depressed mood or dysphoria. An increase in intra-cranial self-stimulation (ICSS) threshold is a commonly used measure of dysphoria in rodents, and the KOR agonists U69,593 ^108^ and salvinorin A [107] have been shown to increase ICSS thresholds in rats; however, another KOR agonist nalfurafine had no effect on this behavior [180]. Note that this effect could be due to the sedative effects of U69,593; however, U69,593 also produced a conditioned place aversion (CPA) in mice [132] and rats [131, 181], an effect consistent with the induction of a negative affective state. The KOR agonist U50,488 also induced a CPA [109], a behavior that we recently showed was exacerbated in chronic pain animals [182]. Given that the vast majority of preclinical studies find that KOR agonism produces depressive-like behaviors (see Table 1), there is strong support for the hypothesis that decreased activity at this receptor underlies the anti-depressant and anti-suicidal properties of buprenorphine.

### Kappa Antagonists

Conversely, KOR antagonists tend to have the opposite effect, producing antidepressant effects in multiple behavioral assays. In particular, a number of studies indicate that KOR antagonists, including norBNI [109, 171, 179, 183-187], JDTic [187], and MCL-144B [188], reduce immobility on the forced swim test in rats and mice. Numerous studies support the anti-depressive effects of KOR anatgonists, particularly norBNI and JDTic (see Table 1), further supporting the role of KOR antagonism in the behavioral effects of buprenorphine.

### Mu Agonists

MOR agonists have been shown to produce antidepressant-like effects, though their efficacy depends on the particular behavioral assay employed (see Table 2). For example, the MOR agonist morphine produced antidepressant-like effects in the learned helplessness model [189] and the tail suspension test [190], but did not have an effect in the forced swim test [171] or in the novelty-induced hypophagia test [175]. Morphine was effective at recovering pain-reduced behaviors including a pain-induced reduction in ICSS responding (increased ICSS threshold) [106] and pain-reduced nesting behavior [191] (although it did not block reduced nesting behavior caused by the KOR agonist U69,593) [191].

### Mu Antagonists

The effects of MOR antagonism on depressive-like behaviors are less conclusive (see Table 2). The selective MOR antagonist cyprodime reduced approach latencies in the novelty induced hypophagia test in mice [175], an antidepressant-like effect which is inconsistent with the hypothesis that a decrease in activity at the MOR should promote depressive behaviors. Furthermore, MOR knockout mice have shown reduced immobility in the forced swim test [192] and reduced anxiogenic and depressive-like responses [193]. These data suggest that activity specifically at the MOR is less likely to underlie the anti-suicidal and antidepressant properties of buprenorphine, since either increases or decreases in activity at the MOR are capable of producing antidepressant behaviors.

Overall, it appears that the evidence for MOR activation underlying buprenorphine’s anti-suicidal effects is less convincing than the evidence for KOR antagonism underlying these effects. This conclusion is based primarily on: 1) the inability of MOR antagonists to block the anti-depressive and anti-suicidal effects of buprenorphine in humans [159, 164, 165] and 2) rodents [173, 174], and 3) the anti-depressive effects of some manipulations which decrease MOR activity [175, 192, 193].

## CONCLUSION

This review has summarized the human and preclinical studies that support further investigation of the potential of buprenorphine treatment for reducing suicidal ideation. The unique pharmacology of buprenorphine that includes partial MOR agonism and KOR antagonism likely contributes to its potential therapeutic effects, where there is strong support for KOR antagonism in alleviating anhedonia and depression. Given the high rate of suicidal ideation and death by suicide in both chronic pain and substance use disorder patients, further research should focus on the development of novel KOR antagonists that do not possess the potential for abuse. Buprenorphine is a potent opioid analgesic (more potent than morphine), and while it is an effective treatment for opioid use disorder, it can be used illicitly where there is evidence of misuse, abuse and diversion [194]. Thus, it is important to understand if drugs which combine buprenorphine with a MOR antagonist (such as naloxone; including drugs like Suboxone®) have anti-suicide properties similar to buprenorphine itself. Overall, buprenorphine and KOR drugs have great potential for the treatment of suicidal behavior and future study may lead to safer and more effective pharmacotherapies.

## Figures and Tables

**TABLE 1 ∣:** Review of evidence supporting the role of KOR in the anti-suicidal effects of buprenorphine. Letters indicate experimental model: (*m*), mouse; (*r*), rat; (*h*), human.

Opioid receptor subtype	Experimental manipulation	Behavioral effect	
KOR	↑,(Up-regulated)	+ (pro-depressant)	***U69,593*** exacerbated pain-depressed ICSS (*r*) [106], elevated ICSS threshold (*r*) [108], depressed nesting behavior (*m*) [191], and produced place aversion (*m, r*) [131, 132, 181] ***Salvinorin A*** increased immobility on forced swim (*r*) 107], elevated ICSS threshold (*r*) [107], and produced psychomimetic effects (*r, h*) [195, 196] ***Cyclazocine*** produced dysphoria & psychomimetic effects (*h*) [197] ***U50,488*** produced conditioned place aversion (CPA) (*m*) [109, 129] and CPA was exacerbated in chronic pain animals (*m*) [182]
		0 (no effect)-(anti-depressant)	***Nalfurafine*** did not alter pain-depressed ICSS (*r*) [180] ***Salvinorin A*** reduced anhedonia caused by chronic mild stress (CMS) (*r*) [198]
	↓,(Down-regulated)	+	
		0	***norBNI*** did not alter pain-depressed ICSS (*r*) [106] or approach latency in the NIH test (*m*) [175] ***JDTic*** did not alter pain-related depression of nesting behavior (*m*) [191] ***norBNI*** reduced immobility on forced swim (*m, r*) [109, 171, 179, 183-187], reduced aversive behaviors produced by inescapable footshock (*m*) [109], reduced expression of learned helplessness (*r*) [199], prevented CPP to gabapentin in a spinal nerve ligation (SNL) injury (*r*) [200], reduced social impairment produced by heroin abstinence (*m*) [201], and attenuated cocaine-withdrawal induced increase in ICSS threshold (*r*) [186] ***JDTic*** reduced immobility on forced swim (*r*) [187], blocked depression of nesting behavior by KOR agonist (*m*) [191], and reduced footshock-induced reinstatement of cocaine seeking (*r*) [187] ***MCL-144B*** reduced immobility on forced swim (*m*) [188] ***KOR knockout mice*** had disrupted BPN-induced reduction in forced swim immobility (*m*) [179] and reduced social aversion following heroin abstinence (*m*) [202]

**TABLE 2 ∣:** Review of evidence supporting the role of MOR in the anti-suicidal effects of buprenorphine. Letters indicate experimental model: (*m*), mouse; (*r*), rat; (*h*), human.

Opioid receptor subtype	Experimental manipulation	Behavioral effect	
MOR	↑	+	
		0	***Morphine*** did not alter behavior on forced swim (*m*) [171], approach latency in NIH test (*m*) [175], nor the depression of nesting behavior by a KOR agonist (*m*) [191]
		-	***Morphine*** reduced learned helplessness (*r*) [189], decreased immobility on the tail suspension test (*m*) [190], blocked pain-depressed ICSS (*r*) 106], and alleviated pain-related depression of nesting behavior (*m*) [191] ***Codeine*** decreased immobility on tail suspension test (*m*) [190, 203] ***Methadone*** reduced learned helplessness (*r*) [204] and decreased immobility on tail suspension test (*m*) [190] ***Tramadol*** reduced learned helplessness (*r*) [204] and decreased immobility on tail suspension test (*m*) [190] ***Opiorphin*** reduced immobility in forced swim (*m*) [205]
	↓	+	***MOR knockout mice*** had disrupted BPN-induced reduction in latency in the NIH test (*m*) [175] ***Mouse model of the OPRM1 A118G polymorphism*** had disrupted BPN-induced reduction in latency in NIH test (*m*) [176] ***OPRM1 A118G polymorphism*** associated with suicidal ideation (*h*) [177] and more severe depression (*h*) [178]
		0	
		-	***Cyprodime*** reduced approach latency in NIH test (*m*) [175] ***MOR knockout mice*** had reduced immobility in forced swim (*m*) [192] and showed reduced anxiogenic and depressive-like responses (*m*) [193]
